# miR-195-5p Reduces VEGFA Expression and Angiogenesis in Colorectal Cancer

**DOI:** 10.3390/ijms27219971

**Published:** 2026-11-06

**Authors:** Emanuele Piccinno, Viviana Scalavino, Giusy Bianco, Serena Mancarella, Ilaria Grassi, Maria Teresa Savino, Raffaele Armentano, Gianluigi Giannelli, Grazia Serino

**Affiliations:** National Institute of Gastroenterology S. De Bellis, IRCCS Research Hospital, Via Turi 27, 70013 Castellana Grotte, BA, Italy; emanuele.piccinno@irccsdebellis.it (E.P.); viviana.scalavino@irccsdebellis.it (V.S.); giusy.bianco@irccsdebellis.it (G.B.); serena.mancarella@irccsdebellis.it (S.M.); ilaria.grassi@irccsdebellis.it (I.G.); maria.savino@irccsdebellis.it (M.T.S.); raffaele.armentano@irccsdebellis.it (R.A.); gianluigi.giannelli@irccsdebellis.it (G.G.)

**Keywords:** miR-195-5p, VEGFA, angiogenesis, colorectal cancer

## Abstract

Angiogenesis is a key process in colorectal cancer (CRC) progression, with vascular endothelial growth factor A (VEGFA) being a major pro-angiogenic mediator. miR-195-5p has previously been identified by our group as a tumor-suppressive miRNA downregulated in CRC. Here, we aimed to investigate whether miR-195-5p contributes to CRC-associated angiogenenic processes through VEGFA regulation. Potential miR-195-5p targets were investigated through bioinformatic predictions and miRNA Target RNA immunoprecipitation. VEGFA expression was evaluated in public CRC datasets and paired tumor/adjacent tissues of a cohort of CRC patients by quantitative real-time PCR and immunohistochemistry. miR-195-5p gain- and loss-of-function experiments were performed in CRC cells to assess VEGFA and VEGFR2 expression and extracellular VEGF-A levels by ELISA. Endothelial tube formation assays using HUVECs were conducted to evaluate paracrine angiogenic effects. VEGFA expression was also analyzed in an AOM/DSS-induced murine CRC model treated with a miR-195-5p mimic. VEGFA was predicted as a conserved miR-195-5p target and was enriched in miR-195-5p-associated RISCs. VEGFA was overexpressed in CRC tissues compared to normal or adjacent non-tumor tissues. miR-195-5p restoration reduced VEGFA mRNA and protein expression, extracellular VEGF-A levels, and VEGFR2 protein levels in the HCT116 CRC cell line, whereas miR-195-5p inhibition produced the opposite molecular profile. Conditioned media from miR-195-5p-overexpressing CRC cells significantly impaired endothelial tube formation. Consistently, miR-195-5p in vivo administration reduced colonic Vegfa expression in AOM/DSS-treated mice. Overall, these findings indicate that miR-195-5p negatively modulates VEGFA expression and reduces the ability of CRC cell-derived conditioned media to support endothelial tube formation. This study extends the tumor-suppressive functions previously attributed to miR-195-5p by supporting its involvement in the regulation of angiogenesis-related processes in CRC.

## 1. Introduction

Colorectal cancer (CRC) is still a major cause of cancer-related mortality despite improvements in screening, surgery, systemic therapy, and molecularly guided treatment. CRC progression and metastatic dissemination represent a wide clinical challenge, and the prognosis for patients with advanced or metastatic disease remains suboptimal [1]. CRC development depends not only on genetic alterations within tumor cells but also on their dynamic interplay with the surrounding tumor microenvironment [2]. Among the critical processes that regulate these close interactions, angiogenesis plays a central role in tumor growth. The process of forming new blood vessels from the existing vasculature supplies oxygen and nutrients to the expanding neoplasm, facilitates its invasion into surrounding tissues, and supports metastatic dissemination. Angiogenesis is a tightly regulated biological process that becomes aberrantly activated in cancer since the balance between pro-angiogenic and anti-angiogenic factors is disrupted [3,4].

The vascular endothelial growth factor (VEGF) family is the best-characterized mediator of tumor angiogenesis and is widely recognized as the predominant pro-angiogenic mediator in solid tumors. VEGFA is a highly conserved dimeric glycoprotein that exists in multiple isoforms generated by alternative splicing, which differ in their bioavailability and interaction with the extracellular matrix, thereby modulating angiogenic signaling [5]. Beyond its canonical mitogenic effects on endothelial cells, VEGFA exerts pleiotropic functions, including the promotion of endothelial cell survival, increased vascular permeability, extracellular matrix remodeling, and modulation of stromal and immune components within the tumor microenvironment [6,7]. Its expression is tightly regulated by hypoxia-inducible factors, particularly HIF-1 [8], as well as by oncogenic pathways such as PI3K/AKT and MAPK/ERK, which are frequently dysregulated in CRC [9,10]. Functionally, VEGFA overexpression in CRC correlates with increased microvessel density, enhanced tumor growth, metastatic dissemination, and poor clinical outcomes [10,11]. Clinically, VEGFA has been extensively validated as a therapeutic target, with anti-VEGF agents demonstrating efficacy in improving progression-free survival in advanced CRC, although resistance mechanisms frequently emerge, underscoring the need for a deeper understanding of its regulatory network [10,12].

The biological effects of VEGFA are primarily mediated through its interaction with the vascular endothelial growth factor receptor 2 (VEGFR2, also known as KDR), the main signaling receptor expressed on endothelial cells and the principal effector of the VEGF pathway. Structurally, VEGFR2 is a receptor tyrosine kinase composed of an extracellular ligand-binding domain, a transmembrane region, and an intracellular kinase domain that contains multiple tyrosine residues and becomes phosphorylated upon activation [13]. Binding of VEGFA induces receptor dimerization and autophosphorylation, leading to the recruitment of adaptor proteins and activation of several downstream signaling cascades [14]. Through these signaling networks, VEGFR2 orchestrates key steps of the angiogenic process, including endothelial cell proliferation, migration, survival, vascular permeability, and the formation of capillary-like networks [13]. In the tumor context, sustained activation of VEGFR2 leads to the development of abnormal, highly permeable, and disorganized vasculature, which contributes to hypoxia, acidosis, and increased interstitial pressure within the tumor mass. These conditions further enhance VEGFA expression, establishing a feedback mechanism that amplifies angiogenic signaling and promotes tumor aggressiveness [15]. In CRC, the VEGFA/VEGFR2 axis is therefore recognized as a central molecular pathway driving tumor-associated angiogenesis and represents a critical target for therapeutic strategies.

In the last few years, emerging evidence has highlighted the contribution of post-transcriptional mechanisms, including microRNA-mediated regulation, in modulating VEGFA expression and angiogenic responses [16]. MicroRNAs (miRNAs) are short non-coding RNAs that act post-transcriptionally as critical regulators of gene expression and influence a broad range of cellular processes, including proliferation, apoptosis, differentiation, invasion, metastasis, immune evasion, and angiogenesis [17,18]. In the context of CRC, miRNAs can play dual roles, acting as either cancer-promoting agents or as inhibitors of tumor development. This dual functionality is influenced by the specific molecular interactions and the cellular environment in which they operate. Their dysregulation can simultaneously influence multiple biological pathways involved in tumor progression and angiogenesis, making miRNAs attractive candidates as both biomarkers and therapeutic targets [19].

Several miRNAs have been shown to directly or indirectly regulate VEGFA expression, thereby critically influencing angiogenic signaling in CRC. Tumor-suppressive miRNAs, such as miR-15a-5p and miR-16-5p, have been reported to directly target VEGFA mRNA, leading to reduced VEGFA expression and impaired angiogenic potential in CRC models. In CRC patients, a consistent association is observed between the reduced expression of the miR-15/16 cluster and heightened angiogenesis, along with a negative prognosis [20,21]. Similarly, circulating miR-20b-5p has been identified as a negative regulator of VEGFA-driven vascular responses in plasma samples from CRC patients. Alterations within the miR-20b/VEGFA axis have been implicated in CRC progression and resistance to anti-angiogenic therapy, highlighting its functional relevance in tumor neovascularization [22,23]. Another well-characterized angiogenesis-regulatory miRNA in CRC is miR-126, whose epigenetic silencing has been associated with increased VEGFA expression, enhanced neovascularization, and invasive tumor behavior. Notably, restoration of miR-126 expression suppresses VEGFA levels and limits tumor-associated angiogenesis, underscoring its tumor-suppressive role in the CRC microenvironment [24,25]. Collectively, these studies underscore miRNA-mediated regulation of VEGFA as a critical molecular layer controlling angiogenic signaling in CRC and support the growing interest in miRNAs as biomarkers and therapeutic modulators of tumor angiogenesis.

In this biological context, miR-195-5p has been identified as a significant tumor-suppressing miRNA in CRC. In our previous studies, miR-195-5p was found to be markedly downregulated in CRC patients and its re-expression consistently suppressed CRC cell growth by limiting proliferative and invasive capacities and by altering cell cycle dynamics and apoptotic cascade [26,27,28,29,30,31]. Importantly, these inhibitory effects were reproduced in vivo, where miR-195-5p restoration markedly reduced tumor burden and impaired colorectal tumor development in chemically induced CRC animal models [27]. These observations identified miR-195-5p as a pleiotropic upstream regulator of tumor aggressiveness in CRC and provided a strong rationale for exploring whether miR-195-5p also influences additional hallmarks of tumor progression, including angiogenic networks.

## 2. Results

### 2.1. Identification of VEGFA as a Candidate Target of miR-195-5p

To unravel the molecular mechanisms and regulatory functions exerted by miR-195-5p in CRC, we carried out a bioinformatic examination to identify potential downstream targets associated with tumor angiogenesis.

The potential interaction between miR-195-5p and VEGFA was explored using four complementary bioinformatic resources, including TargetScan, miRWalk, TarBase, and miRDB. Collectively, these analyses supported VEGFA as a putative target of miR-195-5p. In particular, miRWalk identified 15 predicted miR-195-5p–VEGFA interactions across the analyzed VEGFA transcript variants, with multiple potential binding regions located within the 3′ untranslated region (3′UTR) of VEGFA (Table 1).

Consistent with these findings, TargetScan identified a conserved miR-195-5p recognition site within the VEGFA 3′UTR and predicted sequence complementarity between miR-195-5p and VEGFA transcript (Figure 1A).

To assess whether VEGFA transcripts were associated with RNA-induced silencing complex (RISC), we performed an RNA immunoprecipitation (RIP) employing the miRNA Target IP assay. VEGFA transcripts were significantly enriched in the Ago-associated fraction from cells transfected with miR-195-5p mimic compared with the scrambled-miRNA control (Figure 1B).

Collectively, these findings identify VEGFA as a candidate molecular target of miR-195-5p and support a possible role for this microRNA in the regulation of VEGFA expression in CRC.

### 2.2. VEGFA Is Aberrantly Expressed in Colorectal Cancer

VEGFA plays a central role in driving tumor-associated angiogenesis, so we evaluated its expression profile in human colorectal cancer. The investigation of publicly available transcriptomic datasets demonstrated that VEGFA transcription was significantly upregulated in CRC tissues compared to normal, healthy colonic mucosa (Figure 2A).

These findings indicate that VEGFA is aberrantly expressed in CRC and suggest that increased VEGFA availability may contribute to the pro-angiogenic phenotype associated with colorectal tumor progression. Importantly, the opposite expression pattern previously described for miR-195-5p in CRC supports the hypothesis that the loss of miR-195-5p may contribute to VEGFA dysregulation.

To corroborate these data, we analyzed VEGFA expression in an independent cohort of patients, comparing colorectal tumor lesions with their corresponding matched adjacent normal tissues. Quantitative Real-Time PCR analysis confirmed prominent overexpression of VEGFA mRNA in tumor specimens (Figure 2B). Consistent with these transcriptional findings, immunohistochemical (IHC) analysis revealed stronger VEGF-A staining intensity within the tumor tissue architecture relative to the adjacent non-tumor epithelium (Figure 2C). Moreover, quantitative evaluation of VEGF-A staining further confirmed the increase in protein expression observed in tumor tissues (Figure 2D), confirming the clinical relevance of this pro-angiogenic factor in CRC progression.

### 2.3. miR-195-5p Regulates VEGFA Expression and Indirectly Modulates VEGFR2 in CRC Cells

To investigate the effects of miR-195-5p on the expression of angiogenesis-related molecules, we performed in vitro gain- and loss-of-function experiments using human CRC cells.

HCT116 cells were transiently transfected with either a miR-195-5p mimic or a miR-195-5p inhibitor, and VEGFA mRNA levels were subsequently evaluated by quantitative real-time PCR. After 24 h of transfection, miR-195-5p overexpression resulted in a significant reduction in VEGFA mRNA expression compared with mock-transfected cells (Figure 3A). Conversely, inhibition of endogenous miR-195-5p produced the opposite effect, resulting in increased VEGFA transcript levels in HCT116 cells (Figure 3B).

To further characterize the effects of miR-195-5p on angiogenesis-related molecules, we evaluated the protein levels of VEGF-A and its principal signaling receptor, VEGFR2. Western blot analysis was performed in HCT116 cells following 48 h of transient transfection with miR-195-5p mimic or inhibitor. As shown in Figure 4A, increased miR-195-5p expression markedly reduced VEGFA protein levels in CRC transfected cell lines compared with mock controls. In parallel, VEGFR2 protein expression was also decreased following miR-195-5p mimic transfection, highlighting an indirect consequence of VEGFA suppression. Conversely, inhibition of endogenous miR-195-5p resulted in increased levels of both VEGFA and VEGFR2 proteins (Figure 4B).

These findings demonstrate that modulation of miR-195-5p is associated with reciprocal changes in VEGF-A protein level and extends this effect to VEGFR2.

### 2.4. miR-195-5p Affects Secretion of VEGF-A by CRC Cells

Since VEGF-A is a secreted pro-angiogenic mediator, we next investigated whether the miR-195-5p-dependent regulation of VEGF-A was also reflected in the extracellular compartment. VEGF-A levels were quantified by ELISA in the culture supernatants of CRC cells following transfection with miR-195-5p mimic or inhibitor.

Consistent with the intracellular protein expression data, miR-195-5p overexpression significantly reduced the concentration of VEGF-A in the culture medium of HCT116 cells (Figure 5A). Conversely, miR-195-5p inhibition promoted VEGF-A release, resulting in significantly higher extracellular VEGF-A levels than in control cells (Figure 5B).

These findings indicate that miR-195-5p negatively regulates VEGF-A not only at the cellular level but also affects the amount of VEGF-A released into the extracellular environment, supporting a potential functional impact on VEGFA-dependent paracrine communication.

### 2.5. miR-195-5p Impairs Angiogenic Activity In Vitro

To determine whether the modulation of the VEGFA and VEGFR2 expression induced by miR-195-5p translated into functional effects on angiogenesis, we performed an endothelial tube formation assay using HUVECs exposed to conditioned media collected from transfected CRC cells.

Conditioned media derived from miR-195-5p mimic-transfected cells markedly impaired the formation of capillary-like networks by HUVECs compared with the mock-treated controls. Morphological examination revealed a less organized vascular network characterized by fewer intercellular connections and shorter tubular structures. Quantitative analysis confirmed a significant reduction in total tube length following exposure to conditioned media from cells overexpressing miR-195-5p at both tested concentrations (Figure 6A,B and Supplementary Videos S1–S3).

In contrast, conditioned media obtained from cells transfected with miR-195-5p inhibitor did not induce significant changes in endothelial tube formation compared with control conditions and no statistically significant differences were observed at either 30 or 50 nM inhibitor concentrations (Figure 6C,D and Supplementary Videos S4–S6).

Overall, miR-195-5p overexpression reduced extracellular VEGF-A levels in CRC cell-derived conditioned media and impaired endothelial tube formation.

### 2.6. miR-195-5p Suppresses VEGFA Expression in a Murine Model of Colorectal Carcinogenesis

To evaluate whether the regulatory effect observed in vitro was translated in vivo, we analyzed Vegfa expression in colonic segments obtained from a previously established AOM/DSS-induced mouse model of colorectal carcinogenesis treated with miR-195-5p mimic or vehicle control [27]. Transcriptional profiling of both medial (Figure 7A) and distal (Figure 7B) colonic segments revealed a drastic downregulation of Vegfa mRNA in the cohort of mice systemically treated with the miR-195-5p mimic, in marked contrast to the high expression levels observed in the vehicle-treated control group.

To further validate these molecular changes at the tissue level, we conducted immunofluorescence staining on murine colon sections. The analysis highlighted a strong and widespread VEGF-A positive signal across the neoplastic tissues of vehicle-treated mice. In contrast, both the medial and distal sections derived from animals receiving miR-195-5p therapy exhibited clearly attenuated fluorescence intensity (Figure 8 and Figure S1).

The reduction in VEGF-A expression observed in the colon of miR-195-5p-treated mice was consistent with the in vitro findings and further supports the ability of miR-195-5p to negatively regulate VEGFA in vivo.

## 3. Discussion

CRC progression is characterized by close crosstalk between tumor-intrinsic genetic alterations and the evolving tumor microenvironment [2]. In addition to deregulated proliferation and evasion of apoptosis, tumor growth requires tissue remodeling that enables cancer cells to invade surrounding tissues, engage stromal components and establish a functional vascular network for an adequate blood supply [4]. In this context, angiogenesis emerges as a key process in CRC development, supporting tumor growth, metabolic adaptation, and metastatic spread. This process is primarily driven by VEGFA, a multifunctional regulator of tumor progression that operates into complex signaling networks controlling endothelial cell activation and modulating multiple aspects of tumor biology and clinical outcome [32]. Since VEGFA represents a critical convergence point that integrates multiple regulatory signals, its aberrant expression has been extensively documented across a wide spectrum of malignancies, including colorectal, gastric, lung, ovarian, and hepatocellular carcinomas [33].

In this context, increasing evidence indicates that miRNAs may represent relevant upstream regulators of angiogenic pathways by simultaneously controlling tumor-intrinsic mechanisms and tumor–microenvironment interactions [34,35]. Several miRNAs have been shown to modulate VEGFA expression, highlighting the importance of post-transcriptional regulation in controlling angiogenic mechanisms [36,37]. It has been demonstrated that the restoration of miR-29c expression suppressed gastric cancer cell migration, invasion, and angiogenic activity through the inhibition of the VEGFA/VEGFR2/ERK signaling. These findings suggest that the loss of miR-29c may contribute to the acquisition of a pro-angiogenic phenotype during gastric cancer progression [38]. Similarly, several miRNAs, including miR-140-5p, miR-17-5p, and miR-874-3p, have been shown to reduce VEGFA expression in head and neck cancer models, resulting in attenuation of angiogenesis-associated pathways [39]. In CRC, miR-140-5p downregulation is correlated with an advanced TNM stage since it acts as a crucial tumor suppressor by directly targeting VEGFA, leading to the inhibition of cancer cell proliferation, migration, and invasion [40]. Instead, miR-452 overexpression reduced VEGFA at both the mRNA and protein levels, decreasing extracellular VEGFA availability and attenuating VEGFR2 activation and downstream signaling. The resulting suppression of the VEGFA/VEGFR2 axis was associated with impaired CRC cell proliferation and migration, potentially through the inhibition of SRC/PTK2 and KRAS/BRAF/MAPK pathways [41]. Beyond these direct regulatory interactions, several miRNAs have been reported to indirectly modulate VEGFA expression by targeting upstream regulators of angiogenic signaling. For example, miR-622 suppresses CRC angiogenesis by targeting CXCR4, a positive regulator of VEGFA expression, thereby reducing VEGFA levels and inhibiting endothelial proliferation, migration, and tube formation in conditioned-medium experiments and in a xenograft model [42]. Similarly, the circUBAP2/miR-199a/VEGFA axis has been implicated in CRC progression, with circUBAP2 promoting VEGFA expression by sequestering miR-199a. Conversely, miR-199a overexpression or circUBAP2 depletion reduced VEGFA levels and suppressed CRC growth, further supporting the functional relevance of miR-199a-mediated VEGFA regulation in CRC [43].

Overall, these studies indicate that several miRNAs contribute to the regulation of VEGFA and angiogenesis in CRC, reflecting the complexity of the post-transcriptional control of these processes.

In our previous investigations, we demonstrated a marked downregulation of miR-195-5p in CRC patient cohorts and demonstrated that the restoration of its endogenous levels exerts broad tumor-suppressive effects [27]. Indeed, the gain of miR-195-5p was able to suppress core oncogenic drivers, impairing cellular proliferation, migration, and invasive dynamics while promoting cell cycle arrest [26,28,29,30,31]. Importantly, the therapeutic efficacy of miR-195-5p restoration was confirmed in vivo, since its administration markedly reduced tumor formation and attenuated disease progression in AOM/DSS-treated mice [27]. Mechanistically, our previous studies identified several functional targets of miR-195-5p, including γ-catenin (JUP) [26], a key component of desmosomal junctions that also modulates β-catenin-dependent transcriptional activity, as well as PNN [28], KRT23 [29], and KRT80 [30], which contribute to epithelial and cytoskeletal organization and are involved in tumor growth and dissemination. More recently, we extended the biological role of miR-195-5p to the regulation of apoptotic signaling by demonstrating that its restoration reduces the expression of the anti-apoptotic mediators XIAP, BCL2, and Survivin [31]. This coordinated modulation was associated with increased caspase-3/7 activity, DNA fragmentation, and enhanced apoptosis sensitivity in CRC cells [31]. Together, these findings support a pleiotropic tumor-suppressive role for miR-195-5p across molecular networks controlling cell adhesion, epithelial plasticity, cell-cycle progression, tumor cell survival, and apoptotic resistance.

In the present study, the regulation of VEGFA expression by miR-195-5p broadens this biological framework to include angiogenesis-related processes, further defining the tumor-suppressive functions of miR-195-5p in CRC.

These findings add a microenvironmental component to the multiple tumor-suppressive effects previously attributed to this miRNA. Bioinformatic analysis revealed a conserved miR-195-5p binding site within the VEGFA 3′UTR, while enrichment of VEGFA transcripts in miR-195-5p-associated RISCs supported an association between the two molecules. Although bioinformatic prediction and RISC enrichment support an association between miR-195-5p and VEGFA mRNA, the absence of a reporter assay prevents confirmation of a sequence-specific interaction with the predicted VEGFA 3′UTR binding site.

In parallel, the investigation of public transcriptomic datasets and the molecular analysis performed on an independent series of paired clinical specimens showed that VEGFA was overexpressed in CRC tissues compared with non-tumor colonic mucosa, at both the transcript and protein levels. Considering the reduced miR-195-5p expression previously observed in CRC [27], these opposite expressions suggest that loss of miR-195-5p may contribute to the aberrant accumulation of VEGFA in colorectal tumors. In vitro gain- and loss-of-function experiments in HCT116 cells highlighted that the uptake of miR-195-5p markedly reduced VEGFA mRNA, intracellular protein, and extracellular VEGF-A secretion, while concurrently suppressing VEGFR2 expression. On the other hand, the inhibition of endogenous miR-195-5p led to the opposite molecular pattern. Importantly, the decrease in intracellular VEGF-A was associated with reduced VEGF-A concentrations in cell-culture supernatants, indicating that miR-195-5p also limits the extracellular availability of this pro-angiogenic factor. Consistent with these findings, ELISA analysis confirmed a reduction in VEGFA secretion by CRC cells following miR-195-5p restoration.

The concomitant changes in total VEGFR2 protein levels indicate that miR-195-5p modulation is associated with coordinated changes in multiple angiogenesis-related molecules, although the functional consequences for VEGFA/VEGFR2 signaling remain to be established.

The functional relevance of this regulation by miR-195-5p was also confirmed by the endothelial tube-formation assay. Notably, our data showed that conditioned media from miR-195-5p-overexpressing cells impaired the organization of HUVECs into capillary-like structures and significantly reduced total tube length. Thus, although these findings are consistent with the observed reduction in VEGFA availability, the effect on endothelial cells may also reflect the contribution of other angiogenic factors, as well as the broader tumor-suppressive activity of miR-195-5p in CRC cells. Furthermore, administration of a miR-195-5p mimic in an AOM/DSS mouse model of colorectal carcinogenesis significantly reduced colonic VEGFA expression, supporting the biological activity of miR-195-5p restoration in vivo. However, the reduced VEGFA expression does not necessarily indicate a corresponding reduction in tumor vascularization, which was not directly assessed in the present study.

Given the established role of anti-VEGF therapies in metastatic CRC, the ability of miR-195-5p to suppress VEGFA expression may have potential translational relevance. The present study does not support immediate therapeutic application, but it provides a rationale for investigating whether miR-195-5p restoration could complement currently available VEGF-targeted strategies.

Although the present study provides convergent molecular, functional, clinical, and in vivo evidence supporting the involvement of miR-195-5p in CRC-associated angiogenic processes, some aspects of this regulatory network require further investigation. Additional studies addressing delivery strategies, tissue biodistribution, target specificity, potential off-target effects and safety profiles will be required to determine the translational feasibility of miR-195-5p-based therapeutic approaches. In addition, complementary mechanistic and angiogenic approaches, together with direct evaluation of tumor vascular changes in vivo, may provide a more comprehensive characterization of the role of miR-195-5p in tumor-associated angiogenesis and clarify its potential translational relevance.

Collectively, our findings identify miR-195-5p as an upstream regulator of VEGFA in CRC and show that its restoration reduces VEGFA expression and the pro-angiogenic activity of CRC cell-derived conditioned media. The concomitant modulation of VEGFR2 further suggests that miR-195-5p may affect the VEGFA/VEGFR2 molecules. Overall, these findings expand the molecular functions attributed to miR-195-5p and provide a rationale for further investigating its role in CRC-associated angiogenesis.

## 4. Materials and Methods

### 4.1. Human Colorectal Cancer Tissue Samples

The study included 50 patients with CRC, each contributing a tumor specimen and a matched adjacent non-tumor specimen, for a total of 100 tissue specimens. Twenty patients provided 40 paired fresh/frozen tissue specimens (20 tumors and 20 matched adjacent non-tumor tissues) used for qRT-PCR analysis. A separate cohort of 30 patients provided 60 paired formalin-fixed paraffin-embedded (FFPE) specimens (30 tumors and 30 matched adjacent non-tumor tissues) used for immunohistochemical analysis. Samples were obtained from patients undergoing surgical resection for CRC at the National Institute of Gastroenterology “S. De Bellis”, IRCCS Research Hospital (Castellana Grotte, Bari, Italy). Patients were enrolled according to previously established inclusion criteria, and tumor and adjacent non-tumor specimens were histologically assessed by a pathologist. The study was conducted in accordance with the principles of the Declaration of Helsinki and was approved by the competent Institutional Ethics Review Board (Istituto Tumori Giovanni Paolo II, Bari, Italy, approval number 89/2019). Written informed consent for the use of biological specimens for research purposes was obtained from all participants.

### 4.2. RNA Extraction and Real-Time PCR of FFPE Human Tissue

Total RNA was isolated from formalin-fixed paraffin-embedded (FFPE) colorectal tumor tissues and the corresponding matched adjacent non-tumor tissues using the miRNeasy FFPE Kit (Qiagen, Hilden, Germany), according to the manufacturer’s instructions. Tissue samples were initially deparaffinized using the Deparaffinization Solution (Qiagen, Hilden, Germany) and subsequently subjected to proteinase K digestion and DNase treatment. Purified RNA was eluted in RNase-free water, and its concentration and purity were assessed using a NanoDrop ND-2000 spectrophotometer (Thermo Fisher Scientific, Waltham, MA, USA). Complementary DNA was synthesized from total RNA using the iScript Reverse Transcription Supermix (Bio-Rad Laboratories, Hercules, CA, USA), according to the manufacturer’s protocol. Quantitative real-time PCR was performed using SsoAdvanced Universal SYBR Green Supermix on a CFX96 Real-Time PCR Detection System (Bio-Rad Laboratories, Hercules, CA, USA). Gene-specific primer assays for VEGFA and GAPDH (Bio-Rad Laboratories, Hercules, CA, USA) were used, with GAPDH serving as the endogenous reference gene. Each reaction was performed in triplicate. Relative VEGFA mRNA expression in tumor tissues compared with matched adjacent non-tumor tissues was calculated using the comparative 2^−ΔΔCt^ method.

### 4.3. Immunohistochemistry of VEGFA in FFPE Human Colorectal Cancer Samples

Formalin-fixed paraffin-embedded (FFPE) colorectal tissue specimens were sectioned at a thickness of 3 µm and mounted on Apex Bond IHC slides (Leica Biosystems, Buffalo Grove, IL, USA). Immunohistochemical staining was performed using a BOND III automated immunostainer (Leica Biosystems, Buffalo Grove, IL, USA) with a primary antibody against VEGFA (MA5-13182, Invitrogen, Carlsbad, CA, USA). Sections were subjected to heat-induced antigen retrieval using BOND Epitope Retrieval Solution 2 (Leica Biosystems), based on EDTA buffer. VEGFA immunoreactivity was detected using the Bond Polymer Refine Detection Kit (Leica Biosystems), followed by hematoxylin counterstaining. Images were acquired using the NanoZoomer S20 Digital Slide Scanner (Hamamatsu Photonics, Shizuoka, Japan).

The stained sections were blind-evaluated by a pathologist, who assessed VEGFA expression according to the staining intensity and the extent of positive tissue areas. Immunoreactivity was graded on a four-point scale as follows: 0, negative or absent staining; 1, weak staining; 2, moderate staining; and 3, strong staining.

### 4.4. Cell Culture and miR-195-5p Transfection

Human CRC cell lines HCT116 and human umbilical vein endothelial cells (HUVECs) were obtained from the American Type Culture Collection (ATCC, Manassas, VA, USA). Epithelial colonic cells were maintained in Dulbecco’s Modified Eagle Medium (DMEM; Thermo Fisher Scientific, Waltham, MA, USA) supplemented with 10% heat-inactivated fetal bovine serum (FBS; Thermo Fisher Scientific), 1% penicillin–streptomycin (Thermo Fisher Scientific), 10 mM HEPES, and 1 mM sodium pyruvate (Sigma-Aldrich, St. Louis, MO, USA) whereas endothelial cells were propagated in EndoGRO-LS complete medium supplemented with 2% fetal bovine serum (FBS), 5 ng/mL human recombinant epidermal growth factor (EGF), 50 µg/mL ascorbic acid, 10 mM L-glutamine, 1 µg/mL hydrocortisone hemisuccinate, and 0.75 U/mL heparan sulfate. Cell cultures were established in tissue culture flasks and maintained in a humidified incubator at 37 °C with 5% CO_2_. To ensure the preservation of primary endothelial characteristics and functional stability, HUVECs were strictly utilized between passages 1 and 4 for all functional downstream assays.

For gain- and loss-of-function experiments, HCT116 cells were transiently transfected with synthetic miR-195-5p mimic or miR-195-5p inhibitor molecules (Life Technologies, Carlsbad, CA, USA) at final concentrations of 30 and 50 nM. Transfections were performed using TransIT-TKO Transfection Reagent (Mirus Bio LLC, Madison, WI, USA) according to the manufacturer’s instructions. For each experiment, a mock-transfected condition containing the transfection reagent without miRNA was included as control.

Cells were seeded in 12-well or 6-well plates according to the downstream application. RNA extraction was performed using cells seeded in 12-well plates, whereas protein extraction was performed using cells seeded in 6-well plates, as previously established by our group.

### 4.5. RNA Extraction and Quantitative Real-Time PCR

Total RNA was extracted from cultured cells and mouse colon tissues using TRIzol Reagent (Invitrogen, Thermo Fisher Scientific, Waltham, MA, USA) according to the manufacturer’s instructions. RNA concentration and purity were determined using a NanoDrop ND-2000 spectrophotometer (Thermo Fisher Scientific). For messenger RNA analysis, complementary DNA (cDNA) was synthesized from total RNA using the iScript Reverse Transcription Supermix (Bio-Rad Laboratories, Hercules, CA, USA), according to the manufacturer’s protocol. Quantitative real-time PCR (qRT-PCR) was performed using SsoAdvanced Universal SYBR Green Supermix (Bio-Rad Laboratories, Hercules, CA, USA) on a CFX96 Real-Time PCR Detection System (Bio-Rad Laboratories, Hercules, CA, USA). Gene-specific primers for VEGFA (Bio-Rad Laboratories, Hercules, CA, USA) and GAPDH (Qiagen, Hilden, Germany) were used for the analysis of human samples, while the corresponding murine Vegfa and Gapdh (Bio-Rad Laboratories, Hercules, CA, USA) primers were used for mouse tissues. Relative mRNA expression was calculated using the comparative 2^−ΔΔCt^ method, with GAPDH used as the endogenous reference gene. All experiments were performed using at least three independent biological replicates.

### 4.6. miRNA Target RNA Immunoprecipitation

The recruitment of VEGFA mRNA to Argonaute-containing RISCs following miR-195-5p transfection was assessed using the miRNA Target IP Kit (Active Motif, Carlsbad, CA, USA). This assay is based on the immunoprecipitation of Argonaute-containing RNA-induced silencing complexes (RISCs), allowing the detection of endogenous mRNA transcripts associated with the miRNA machinery. Briefly, cells were lysed using the reagents supplied with the kit, and miRNA/mRNA-containing RISCs were immunoprecipitated using the pan-Argonaute antibody included in the kit. A nonspecific IgG antibody was used as a negative immunoprecipitation control. For the comparison shown in Figure 1B, cells transfected with scrambled miRNA served as the negative miRNA control for the miR-195-5p mimic condition. Following immunoprecipitation, RNA associated with the precipitated complexes was purified according to the manufacturer’s instructions and subjected to reverse transcription and qRT-PCR analysis. VEGFA transcript enrichment was determined by comparison with the IgG control.

### 4.7. Protein Extraction and Western Blotting

Total cellular proteins were extracted using the T-PER Tissue Protein Extraction Reagent (Thermo Fisher Scientific) supplemented with a protease inhibitor cocktail (Sigma-Aldrich, St. Louis, MO, USA). Protein concentration was determined using the Bradford assay (Bio-Rad Laboratories, Hercules, CA, USA).

Equal amounts of protein were denatured in Laemmli sample buffer at 100 °C for 5 min and separated by SDS-PAGE using 4–20% TGX Stain-Free Precast Gels (Bio-Rad Laboratories, Hercules, CA, USA). Proteins were subsequently transferred onto 0.2 μm polyvinylidene difluoride (PVDF) membranes using the Trans-Blot Turbo Transfer System (Bio-Rad Laboratories, Hercules, CA, USA), according to the manufacturer’s instructions.

Membranes were incubated with primary antibodies directed against VEGFA (A14925, Antibodies, Cambridge, UK) and VEGFR2 (#2479, Cell Signaling Technology, Danvers, MA, USA) followed by the appropriate horseradish peroxidase (HRP)-conjugated secondary antibodies in an automated iBind Flex Western Device (Thermo Fisher Scientific, Waltham, MA, USA). Goat Anti-mouse IgG-(H + L)-HRP conjugate (170-6516, Biorad Laboratories, CA, USA) and Goat Anti-rabbit IgG-(H + L)-HRP conjugate (31466, Invitrogen, Carlsbad, CA, USA) were used. β-Tubulin levels were detected as a loading control (sc-166729, Santa Cruz Biotechnology, Inc., Heidelberg, Germany).

Immunoreactive bands were visualized using a ChemiDoc Imaging System (Bio-Rad Laboratories), and densitometric analysis was performed using the ImageJ software 1.54d. Protein expression levels were normalized to the corresponding loading control.

### 4.8. Extracellular VEGFA Secretion

To determine extracellular VEGFA levels, conditioned culture media were collected from HCT116 cells following transfection with the miR-195-5p mimic or inhibitor. Cell culture supernatants were collected under the same experimental conditions and clarified by centrifugation to remove the cellular debris. Conditioned media were collected 48 h after transfection. Total protein concentration was determined separately in each supernatant, and equivalent amounts of total protein from each condition were used for VEGF-A quantification. This normalization reduces differences in sample loading but does not fully exclude effects of miR-195-5p treatment on cell number or viability.

VEGFA concentrations were quantified using a human DuoSet ELISA (R&D Systems, Minneapolis, MN, USA) according to the manufacturer’s instructions. Briefly, the assay was performed in 96-well microplates (Costar Corning, Corning, NY, USA) previously coated with the specific capture antibody. Following sample loading and incubation, VEGFA bound to the immobilized antibody was detected using the corresponding biotinylated detection antibody. Streptavidin-conjugated to horseradish peroxidase (HRP) was subsequently added to generate the enzymatic detection complex. After appropriate washing steps, the signal was developed by the addition of 3,3′,5,5′-tetramethylbenzidine (TMB; Abcam, Cambridge, UK). The enzymatic reaction was stopped according to the manufacturer’s instructions, and absorbance was measured at 450 nm using a SPECTROstar Omega microplate reader (BMG Labtech, Ortenberg, Germany). VEGFA concentrations were calculated from a standard curve generated using the VEGFA standards provided with the assay kit.

### 4.9. Endothelial Tube-Formation Assay

The angiogenic activity of CRC cell-derived conditioned media was evaluated using an in vitro endothelial tube-formation assay. HCT116 cells were transfected with miR-195-5p mimic or inhibitor, and conditioned media were collected after 48 h under standardized culture conditions.

For tube-formation assays, HUVECs were seeded onto Matrigel-coated 96-well plates at a density of 1.4 × 10^4^ cells/well. Conditioned media were concentrated and normalized according to total protein content before being added to HUVEC cultures. The volume of conditioned medium was adjusted to obtain equivalent final protein concentrations (10 μg/mL), and EndoGRO medium was added to a final volume of 80 μL. Tube formation was monitored by time-lapse microscopy over a 16-h period. All wells were automatically acquired every 15 min using an Eclipse Ti2 microscope (Nikon Inc., Melville, NY, USA). Tube formation parameters were quantified using ImageJ software.

### 4.10. Immunofluorescence in Mouse Samples

The in vivo effects of miR-195-5p on Vegfa expression were evaluated by immunofluorescence using colon tissues derived from a previously established AOM/DSS-induced mouse model of colorectal carcinogenesis. The animal procedures, treatment schedule, ethical approvals, and experimental design have been described in detail in our previous publication [27].

Colon specimens were fixed in formalin and embedded in paraffin. Sections were deparaffinized and subjected to antigen retrieval in citrate buffer (pH 6.0) at 98 °C for 30 min. Sections were permeabilized with PBS containing 0.5% Triton X-100 and blocked using BlockAid Blocking Buffer (Invitrogen, Carlsbad, CA, USA) for 1 h at room temperature.

Sections were incubated overnight at 4 °C with a primary antibody against VEGFA (A14925, Antibodies, Cambridge, UK). After washing, the sections were incubated for 1 h with the corresponding Alexa Fluor-conjugated secondary antibody (Thermo Fisher Scientific, Waltham, MA, USA). Nuclei were counterstained using VECTASHIELD Antifade Mounting Medium containing DAPI (Vector Laboratories, Newark, CA, USA).

Fluorescence images were acquired using a ZEISS Celldiscoverer 7 imaging system (Zeiss, Jena, Germany). Images were collected using the appropriate fluorescence channels and a 10× objective, and the single channels were subsequently merged for analysis.

### 4.11. Bioinformatic and Statistical Analysis

The potential interaction between miR-195-5p and VEGFA was investigated using TargetScan [44], miRWalk [45], TarBase [46] and miRDB [47] prediction algorithms.

For the analysis of VEGFA expression in human CRC, publicly available transcriptomic datasets were interrogated using the UALCAN web-based platforms [48]. VEGFA expression levels in CRC tissues were compared with those detected in normal colorectal tissues according to the datasets available in each platform.

All experimental data were obtained from at least three independent biological replicates and are reported as mean ± SEM. Statistical analyses were performed using GraphPad Prism 9 software. Differences between two experimental groups were evaluated using a two-tailed Student’s *t*-test. A *p*-value < 0.05 was considered statistically significant.

## 5. Conclusions

In conclusion, this study expands the previously described tumor-suppressive activity of miR-195-5p by supporting its involvement in VEGFA regulation and angiogenesis-related processes.

Restoration of miR-195-5p reduced VEGFA expression at both transcript and protein levels, limited its extracellular availability, and was associated with reduced VEGFR2 expression in CRC cells. In addition, miR-195-5p overexpression restrained the ability of CRC cells to induce endothelial tube formation and significantly reduced colonic VEGFA expression in vivo. Overall, these findings highlighted the involvement of miR-195-5p in VEGFA regulation and further support its broad antitumor activity in CRC.

## 6. Patents

An Italian patent entitled “MiRNA or combination of miRNAs for therapy” was issued on 4 June 2024 to the Ente Ospedaliero Specializzato in Gastroenterologia “Saverio de Bellis”.

## Figures and Tables

**Figure 1:**
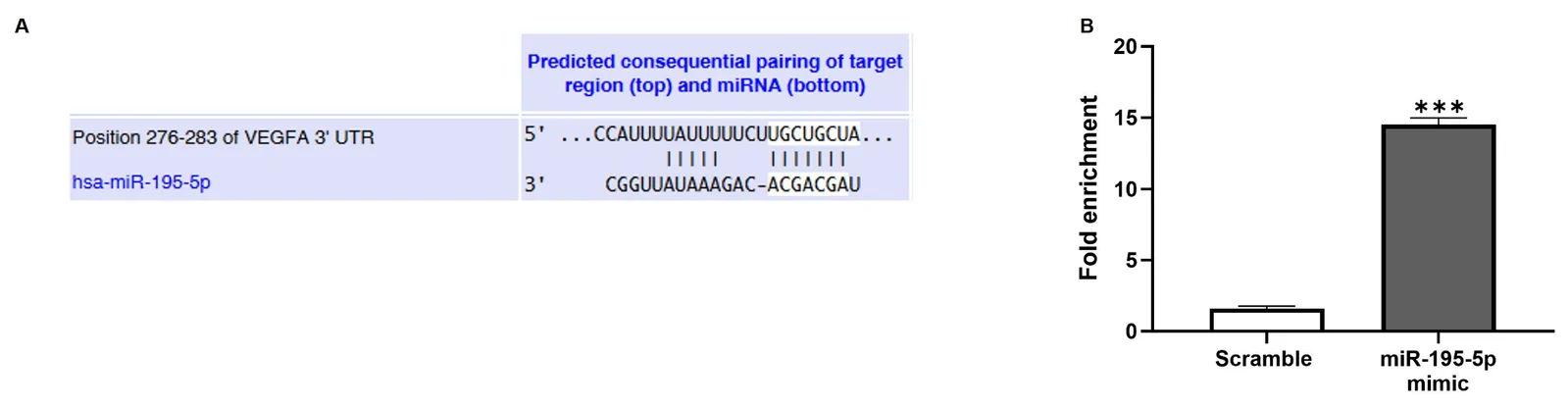
Bioinformatic prediction and RNA immunoprecipitation (RIP)-based assessment of VEGFA association with miR-195-5p. (**A**) TargetScan-predicted sequence complementarity between miR-195-5p and the recognition site located within the VEGFA 3′UTR. (**B**) RNA immunoprecipitation analysis using the miRNA Target IP assay showing the enrichment of VEGFA mRNA in miR-195-5p-associated complexes. Data are presented as mean ± SEM from three independent experiments. *** *p* < 0.0001.

**Figure 2:**
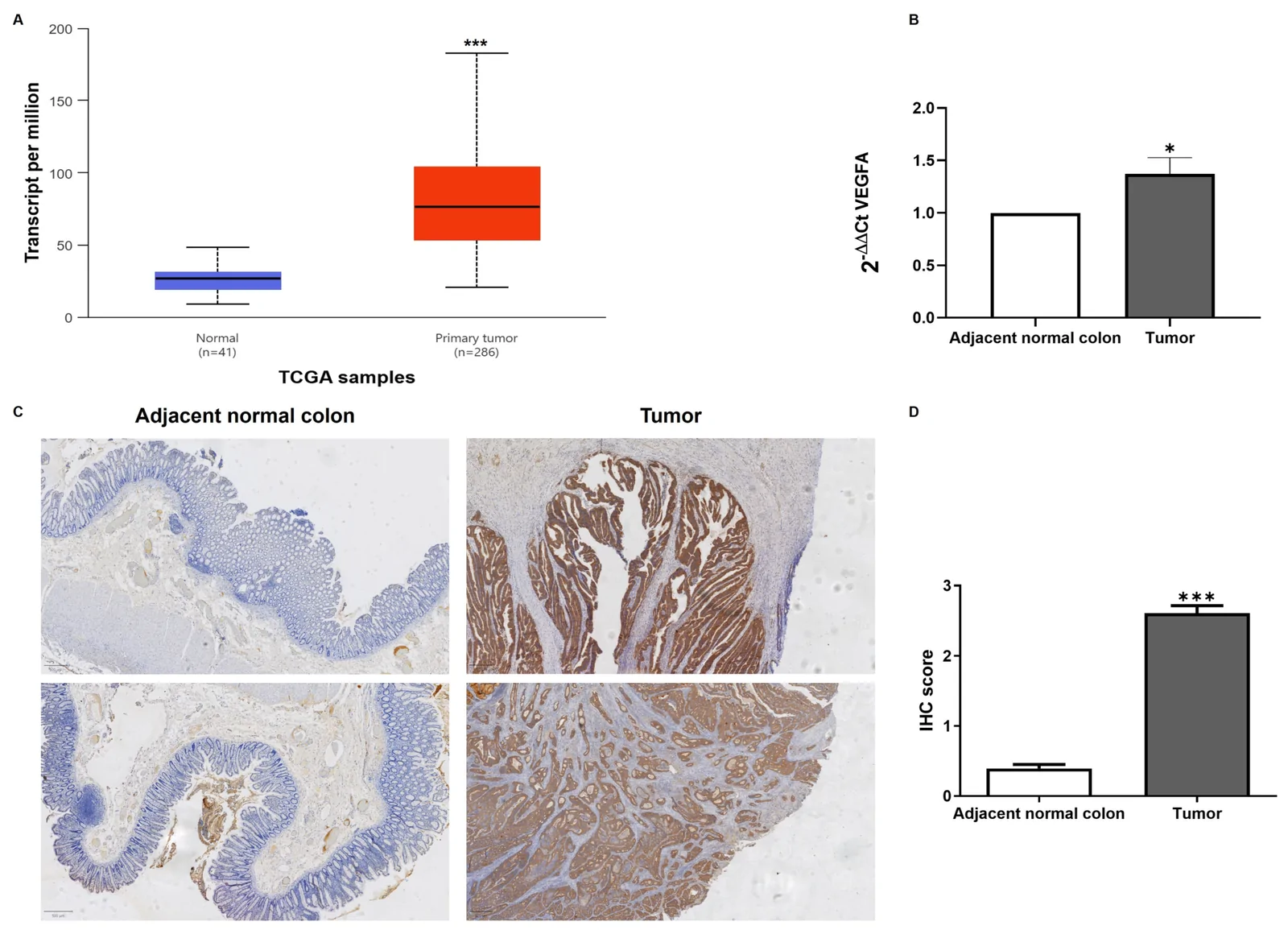
VEGFA expression in CRC. (**A**) VEGFA mRNA expression in colorectal cancer tissues compared with normal colorectal tissues according to publicly available transcriptomic datasets analyzed using the UALCAN tool. (**B**) VEGFA mRNA expression assessed by quantitative real-time PCR in tumor tissues and matched adjacent non-tumor tissues from 20 patients with CRC (*n* = 40). Relative expression was calculated using the 2^−ΔΔCt^ method, with GAPDH as the endogenous reference gene. (**C**) Representative immunohistochemical staining showing VEGFA expression in colorectal tumor and matched adjacent non-tumor tissues from a separate cohort of 30 patients (60 FFPE specimens in total). Acquisition at 4× magnification (**D**) Quantitative analysis of VEGF-A immunoreactivity in healthy and diseased tissues. VEGF-A expression was evaluated according to staining intensity using an immunoreactivity score (*n* = 60). Sections were evaluated by a pathologist blinded to sample identity. * *p* < 0.05, *** *p* < 0.0001.

**Figure 3:**
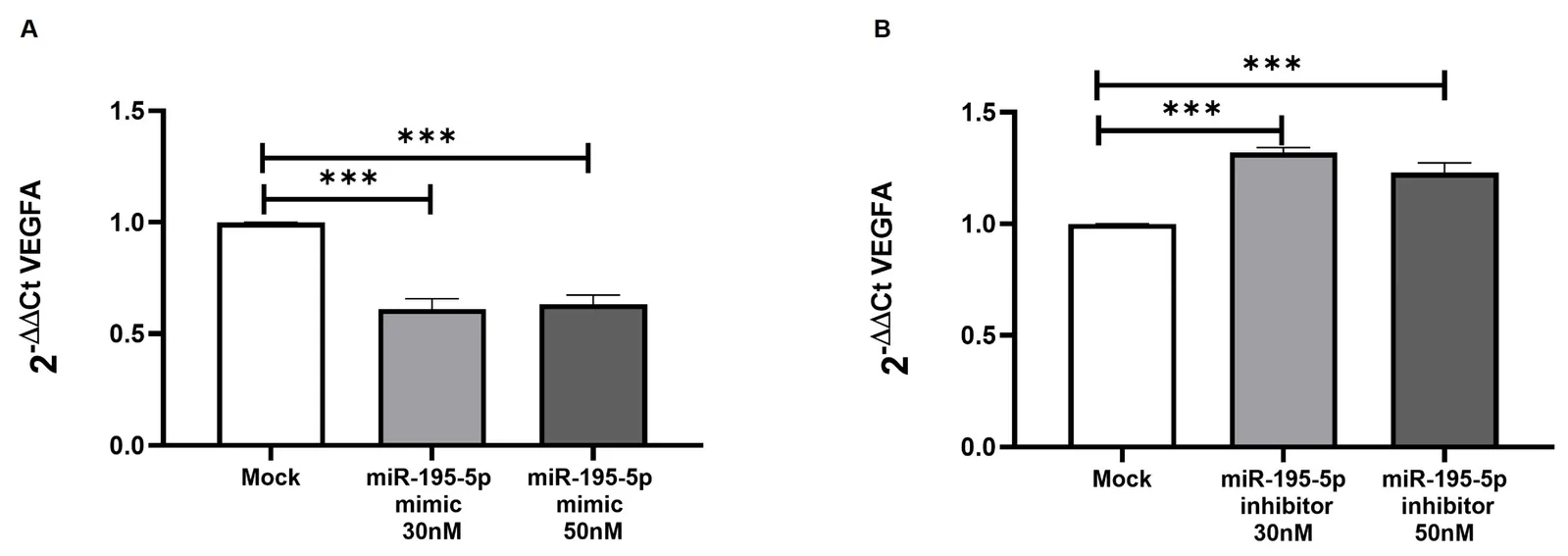
miR-195-5p modulates VEGFA expression at the transcriptional level in CRC cells. VEGFA mRNA expression in HCT116 cells following transient transfection with (**A**) miR-195-5p mimic or (**B**) miR-195-5p inhibitor at the indicated concentrations. VEGFA expression was measured by quantitative real-time PCR and calculated using the 2^−ΔΔCt^ method, with GAPDH as the endogenous reference gene. Data are presented as mean ± SEM from at least three independent experiments. Statistical significance was determined using a two-tailed Student’s *t*-test relative to the corresponding mock control. *** *p* < 0.0001.

**Figure 4:**
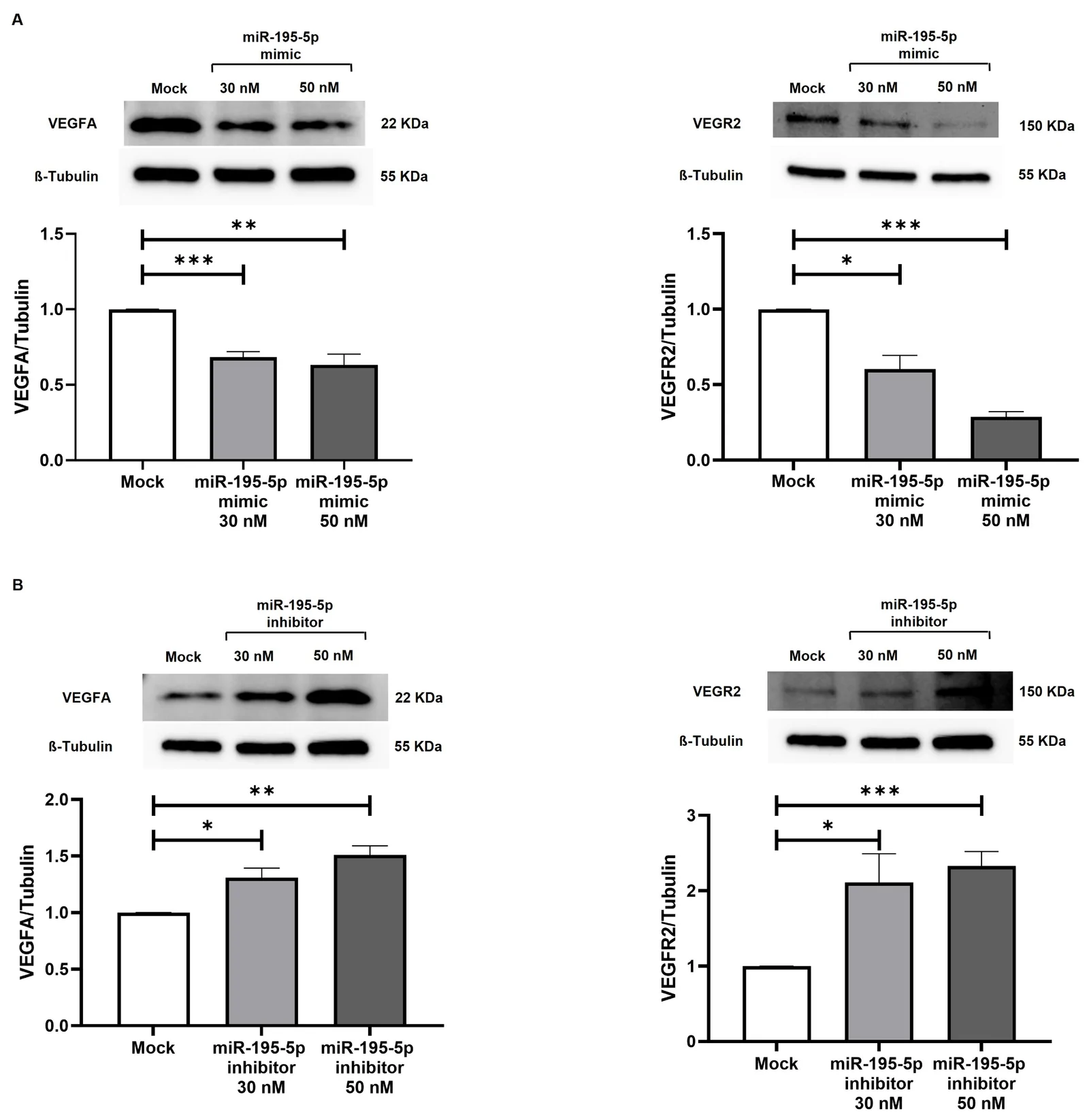
miR-195-5p affects VEGF-A and VEGFR2 protein levels. Western blot analysis of VEGFA and VEGFR2 protein expression following transfection with miR-195-5p mimic (**A**) or miR-195-5p inhibitor (**B**) molecules. miR-195-5p overexpression at 30 nM and 50 nM concentrations reduced VEGF-A and VEGFR2 protein levels compared with mock-transfected cells, whereas miR-195-5p suppression had the opposite effect. β-Tubulin was used as a loading control. Densitometric values were normalized to β-tubulin and expressed relative to the corresponding mock control. Data are shown as mean ± SEM from at least 3 independent experiments. * *p* < 0.05; ** *p* < 0.001; *** *p* < 0.0001.

**Figure 5:**
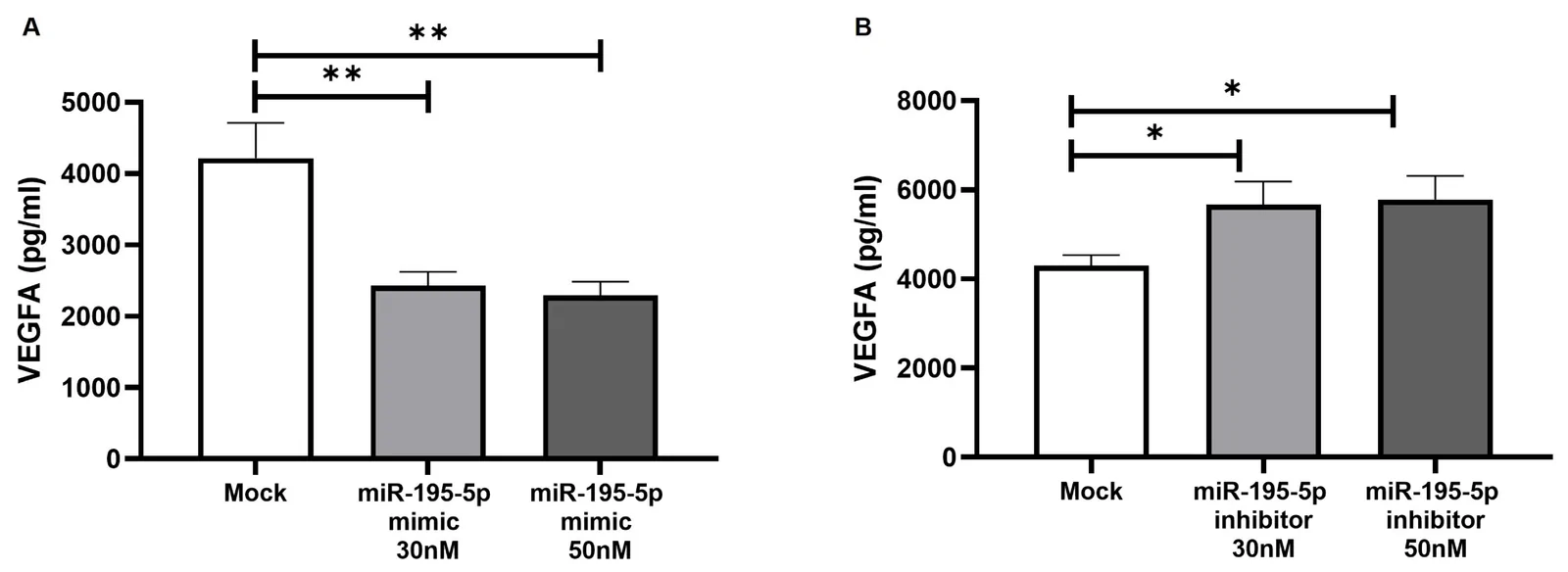
miR-195-5p controls extracellular VEGF-A secretion. Extracellular VEGF-A levels were determined by ELISA in conditioned media collected from HCT116 cells after 48 h of transfection. (**A**) VEGF-A concentrations following miR-195-5p gain at 30 and 50 nM. (**B**) VEGFA concentrations after inhibition of endogenous miR-195-5p levels. VEGF-A levels were quantified in the conditioned medium. Supernatants were normalized to total protein content before VEGF-A quantification. Data are presented as mean ± SEM from independent experiments. * *p* < 0.05, ** *p* < 0.01.

**Figure 6:**
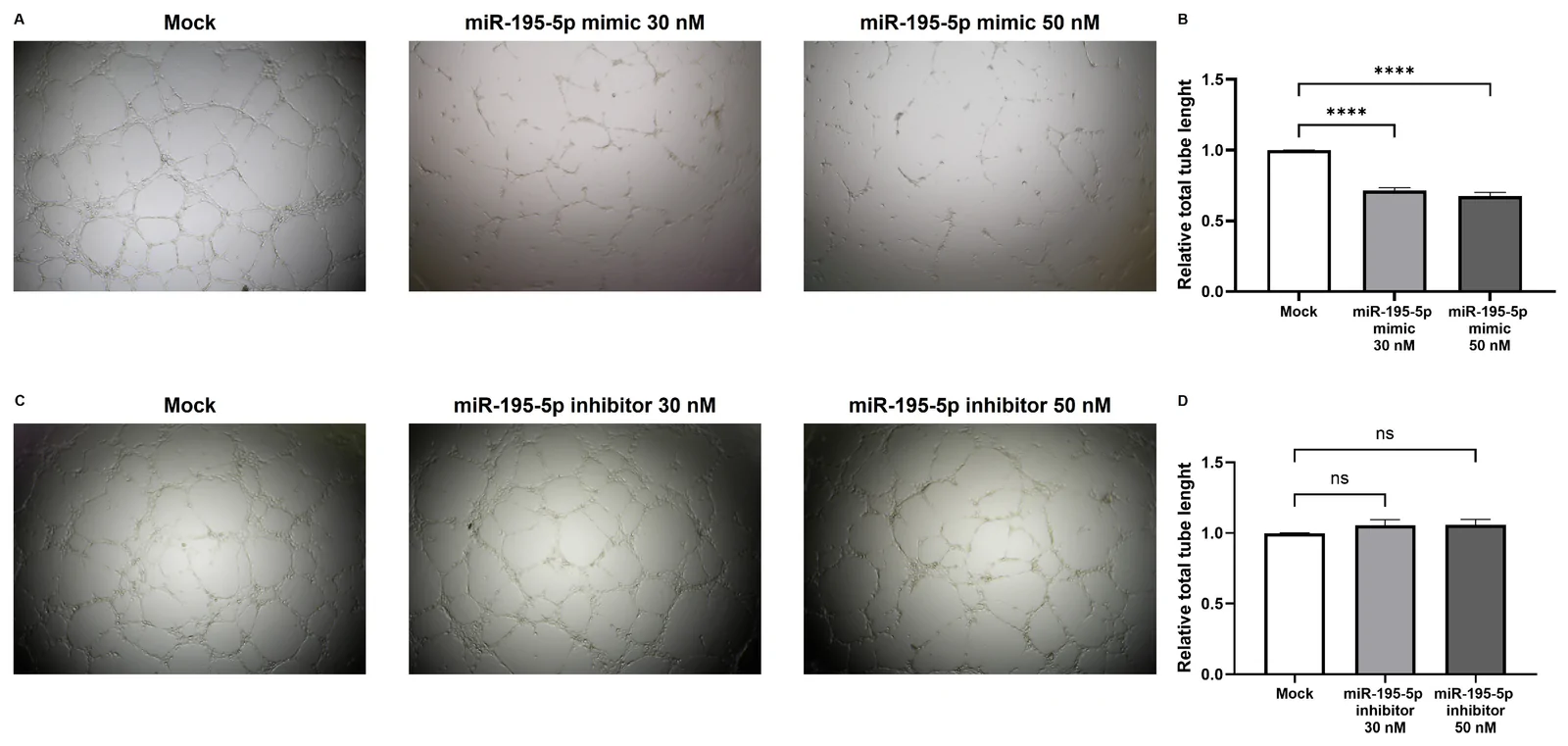
miR-195-5p restrained the angiogenic activity of CRC cells. (**A**) Representative phase-contrast images of HUVEC tube formation following exposure to conditioned media collected from HCT116 cells transfected with miR-195-5p mimic. (**B**) Quantification of total tube length under the experimental conditions shown in panel A. (**C**,**D**) Effect of miR-195-5p inhibition on HUVEC tube formation and relative quantification. Total tube length was quantified using ImageJ software v.1.54d. Representative images and quantitative data are presented as mean ± SEM from at least three independent experiments. **** *p* < 0.0001; ns stands for not significant.

**Figure 7:**
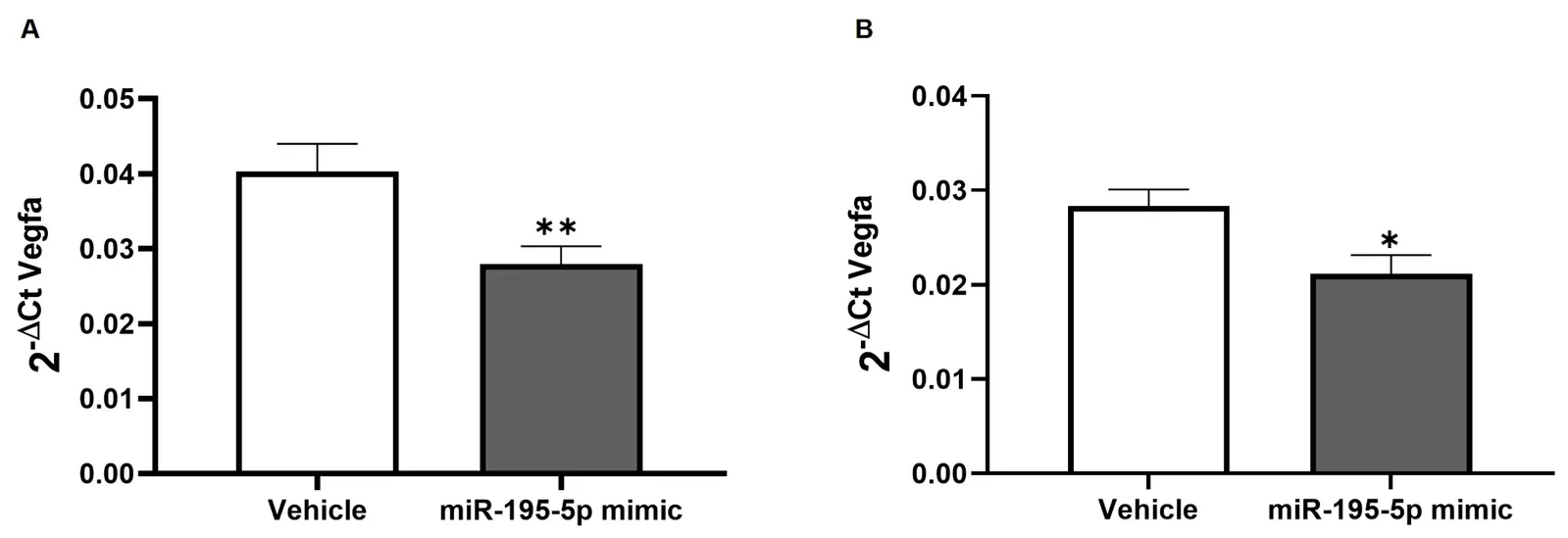
miR-195-5p lowered Vegfa mRNA expression in the colon of AOM/DSS-treated mice. *Vegfa* mRNA expression was evaluated by quantitative real-time PCR in (**A**) medial and (**B**) distal colon segments collected from AOM/DSS-treated mice administered either vehicle or miR-195-5p mimic. Relative expression was calculated using the 2^−ΔΔCt^ method, with *Gapdh* as the endogenous reference gene. Data are presented as mean ± SEM. Statistical significance was determined using a two-tailed Student’s *t*-test. * *p* < 0.05; ** *p* < 0.01.

**Figure 8:**
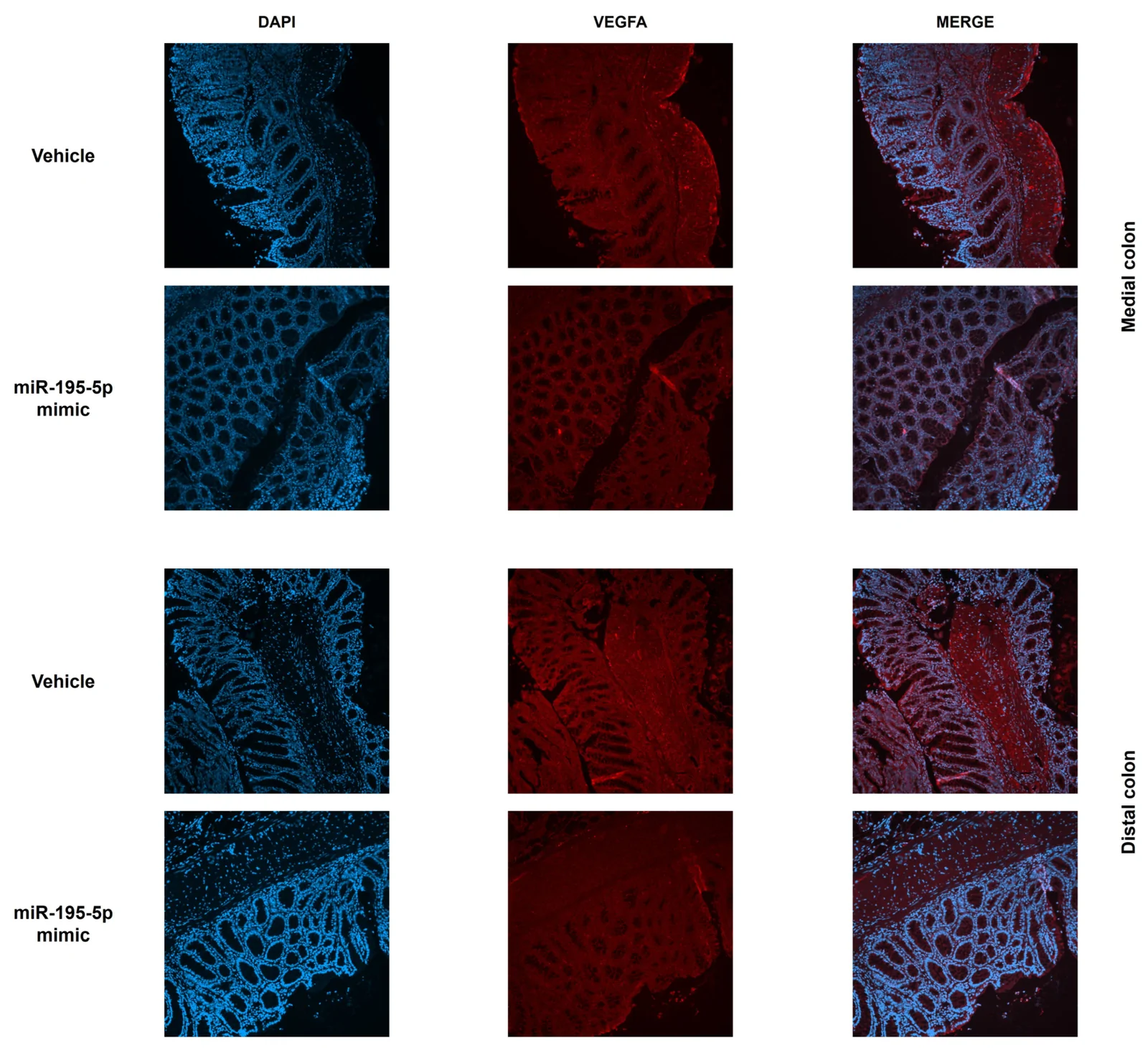
In vivo effect of miR-195-5p on VEGF-A protein expression. VEGF-A protein expression was evaluated in the medial and distal colon of AOM/DSS-treated mice (*n* = 14 mice per group). Administration of miR-195-5p reduced VEGF-A protein levels, consistent with the corresponding gene expression data. Representative images of VEGF-A immunofluorescence and DAPI nuclear staining, together with the corresponding merged images, are shown. Original magnification: 20×.

**Table 1:** miRWalk analysis showing predicted miR-195-5p-binding regions within the 3′UTR of 15 VEGFA transcript variants.

miRNA ID	Refseq ID	Gene Symbol	Score	Au	Me	N Pairings	Binding Region Length	Longest Consecutive Pairings	Position
hsa-miR-195-5p	NM_001171623	VEGFA	1	0.6	−4.99396	19	25	8	3UTR
hsa-miR-195-5p	NM_001171624	VEGFA	1	0.6	−4.99396	19	25	8	3UTR
hsa-miR-195-5p	NM_001171625	VEGFA	1	0.6	−4.99396	19	25	8	3UTR
hsa-miR-195-5p	NM_001171626	VEGFA	1	0.6	−4.99396	19	25	8	3UTR
hsa-miR-195-5p	NM_001171627	VEGFA	1	0.6	−4.99396	19	25	8	3UTR
hsa-miR-195-5p	NM_001171628	VEGFA	1	0.6	−4.99396	19	25	8	3UTR
hsa-miR-195-5p	NM_001171629	VEGFA	1	0.6	−4.99396	19	25	8	3UTR
hsa-miR-195-5p	NM_001171630	VEGFA	1	0.6	−4.99396	19	25	8	3UTR
hsa-miR-195-5p	NM_003376	VEGFA	1	0.6	−4.99396	19	25	8	3UTR
hsa-miR-195-5p	NM_001025366	VEGFA	1	0.6	−4.99396	19	25	8	3UTR
hsa-miR-195-5p	NM_001025367	VEGFA	1	0.6	−4.99396	19	25	8	3UTR
hsa-miR-195-5p	NM_001025368	VEGFA	1	0.6	−4.99396	19	25	8	3UTR
hsa-miR-195-5p	NM_001025369	VEGFA	1	0.6	−4.99396	19	25	8	3UTR
hsa-miR-195-5p	NM_001025370	VEGFA	1	0.6	−4.99396	19	25	8	3UTR
hsa-miR-195-5p	NM_001033756	VEGFA	1	0.6	−4.99396	19	25	8	3UTR

## Data Availability

Data are within the article, Supplementary Materials Raw data of Western blot experiments presented in the study are openly available in FigShare at doi 10.6084/m9.figshare.33729667.

## Supplementary Materials

The following supporting information can be downloaded at https://www.mdpi.com/article/10.3390/ijms27219971/s1.
