# Supplementary material for: miR-195-5p Reduces VEGFA Expression and Angiogenesis in Colorectal Cancer
Source: Int J Mol Sci. 2026 Nov 6;27(21):9971. doi: 10.3390/ijms27219971 (PMC13764365; doi:10.3390/ijms27219971)
Supplement: Supplementary file 1 [file ijms-27-09971-s001.zip › Figure S1.pdf]

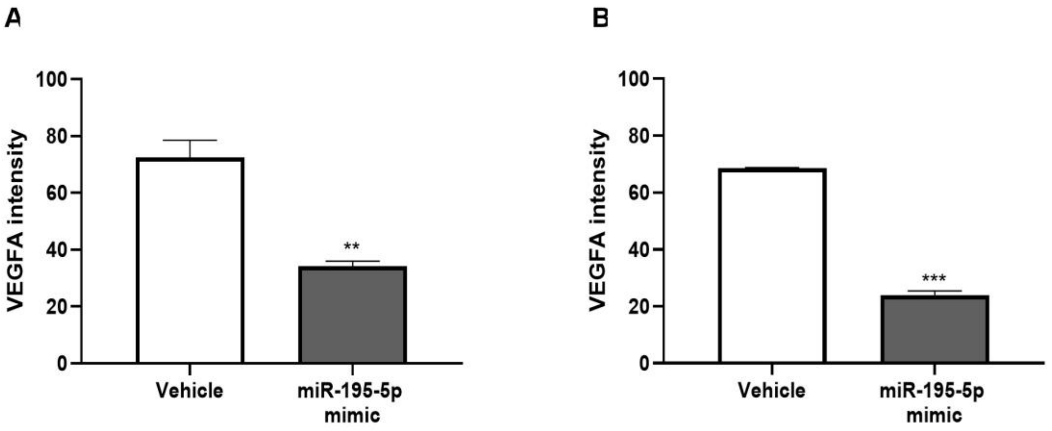

Figure S1. miR-195-5p reduced VEGF-A expression in the colon of AOM/DSS-treated mice. VEGF-A expression was evaluated by immunofluorescence in (**A**) medial and (**B**) distal colon segments collected from AOM/DSS-treated mice receiving administered either vehicle or miR-195-5p mimic.
